# Propiedades del CP-QOL-PCQ para medir calidad de vida relacionada con la salud en niños, niñas y adolescentes con parálisis cerebral en Argentina

**DOI:** 10.31053/1853.0605.v80.n3.40042

**Published:** 2023-09-29

**Authors:** Silvina Berra, Natalia Herrera Sterren, Javier Sánchez Rosas

**Affiliations:** 1 Universidad Nacional de Córdoba. Facultad de Ciencias Médicas. Consejo Nacional de Investigaciones Científicas y Técnicas Argentina; 2 Universidad Nacional de Córdoba. Consejo Nacional de Investigaciones Científicas y Técnicas Argentina; 3 Universidad Católica de Temuco, Facultad de Ciencias de la Salud, Departamento de Psicología Chile

**Keywords:** parálisis cerebral, niños con discapacidad, adolescencia, encuestas y cuestionarios, confiabilidad y validez, cerebral palsy, disabled children, adolescence, surveys and questionnaires, reliability and validity, paralisia cerebral, crianças com deficiência, adolescência, inquéritos e questionários, confiabilidade e validade

## Abstract

**Introducción:**

El impacto de la parálisis cerebral (PC) en la calidad de vida exige la incorporación de valoraciones subjetivas de la salud, el estado funcional y el bienestar. Una medida de calidad de vida relacionada con la salud (CVRS) específica para PC es el cuestionario australiano CP-QOL, cuya versión para cuidadores (PCQ) fue adaptada en Argentina. Este estudio se propuso lograr la estructura final y evaluar la fiabilidad y validez del CP-QOL-PCQ para población infantil y adolescente argentina.

**Métodos:**

Participaron 100 cuidadores de personas de 3 a 24 años con diagnóstico de PC de Argentina. Además del CP-QOL-PCQ se aplicó el cuestionario KIDSCREEN-27. Se realizaron análisis de distribuciones de respuesta de todos los ítems, correlaciones inter-ítems y consistencia interna de las escalas mediante el alfa de Cronbach. Se probó la validez convergente del instrumento mediante la correlación con escalas de similar contenido del KIDSCREEN y mediante hipótesis a priori en grupos de diferente edad y afectación de la funcionalidad.

**Resultados:**

Se logró una estructura final del CP-QOL-PCQ con 8 escalas multidimensionales, con satisfactorias correlaciones inter-ítems satisfactorias (>0.30) y consistencia interna (>0.70). Se obtuvieron correlaciones moderadas y altas (r>0.30) entre dimensiones similares del CP-QOL-PCQ y del KIDSCREEN con conceptos similares. Se confirmaron mayores puntuaciones de CVRS a menor edad y a menor nivel de afectación funcional (d de Cohen >0,20).

**Conclusión:**

Este trabajo aporta evidencias de fiabilidad y validez esperables para un instrumento de medición de la CVRS que puede ser implementado en población infantil y adolescente argentina con PC.

## Abstract (en)

**Introduction:**

The impact of cerebral palsy (CP) on quality of life requires the incorporation of subjective assessments of health, functional status, and well-being. A specific health-related quality of life (HRQoL) measure for CP is the Australian CP-QOL questionnaire, whose version for caregivers (PCQ) was adapted in Argentina. This study aimed to achieve the final structure and evaluate the reliability and validity of the CP-QOL-PCQ for the Argentine child and adolescent population.

**Methods:**

100 caregivers of people from 3 to 24 years old with a diagnosis of CP from Argentina participated. In addition to the CP-QOL-PCQ, the KIDSCREEN-27 questionnaire was applied. Analysis of response distributions of all items, inter-item correlations, and internal consistency of the scales were performed using Cronbach's alpha. The convergent validity of the instrument was tested through the correlation with scales of similar content from the KIDSCREEN and through a priori hypotheses in groups of different ages and functional impairment.

**Results:**

A final structure of the CP-QOL-PCQ was achieved with 8 multidimensional scales, with satisfactory inter-item correlations (>0.30) and internal consistency (>0.70). Moderate and high correlations (r>0.30) were obtained between similar dimensions of the CP-QOL-PCQ and the KIDSCREEN with similar concepts. Higher HRQoL scores were confirmed at a younger age and at a lower level of functional impairment (Cohen's d >0.20).

**Conclusion:**

This provides evidence of reliability and validity to be expected for a HRQoL measurement instrument that can be implemented in the Argentine child and adolescent population with CP.

## Resumo

**Introdução:**

O impacto da paralisia cerebral (PC) na qualidade de vida requer a incorporação de avaliações subjetivas de saúde, estado funcional e bem-estar. Uma medida de qualidade de vida relacionada à saúde (QVRS) específica para PC é o questionário australiano CP-QOL, cuja versão para cuidadores (PCQ) foi adaptada na Argentina. Este estudo teve como objetivo alcançar a estrutura final e avaliar a confiabilidade e validade do CP-QOL-PCQ para a população infanto-juvenil argentina.

**Métodos:**

participaram 100 cuidadores de pessoas de 3 a 24 anos com diagnóstico de PC da Argentina. Além do CP-QOL-PCQ, foi aplicado o questionário KIDSCREEN-27. A análise das distribuições de resposta de todos os itens, correlações entre itens e consistência interna das escalas foram realizadas usando o alfa de Cronbach. A validade convergente do instrumento foi testada por meio da correlação com escalas de conteúdo semelhante do KIDSCREEN e por hipóteses a priori em grupos de diferente idade e comprometimento da funcionalidade.

**Resultados:**

Obteve-se uma estrutura final do CP-QOL-PCQ com 8 escalas multidimensionais, com correlações entre itens satisfatórias (>0,30) e consistência interna (>0,70). Correlações moderadas e altas (r>0,30) foram obtidas entre dimensões semelhantes do CP-QOL-PCQ e do KIDSCREEN com conceitos semelhantes. Maiores escores de QVRS foram confirmados em menor idade e em menor nível de comprometimento funcional (d de Cohen >0,20).

**Conclusão:**

Este trabalho fornece evidências de confiabilidade e validade esperáveis para um instrumento de medição de QVRS que pode ser implementado na população infantil e adolescente argentina com PC.

**CONCEPTOS CLAVE**

Qué se sabe sobre el tema: Los indicadores de calidad de vida relacionada con la salud incorporan una valoración subjetiva del bienestar en múltiples dimensiones que son relevantes para la persona. Sus instrumentos de medición deben ser lingüística y culturalmente adecuados, específicos a la edad y condición del sujeto, factibles de implementar y ofrecer información con probada fiabilidad y validez. Qué aporta este trabajo: Se presenta la versión del CP-QOL-PCQ para medir calidad de vida relacionada con la salud en niños, niñas y adolescentes con parálisis cerebral en Argentina, su estructura, modelo y evidencias de fiabilidad y validez para que pueda ser aplicado en el ámbito clínico, escolar y en futuras investigaciones.

**Divulgación**

El CP-QOL-PCQ es una herramienta para valorar la calidad de vida relacionada con la salud en niños, niñas y adolescentes con parálisis cerebral en Argentina. La aplicación de este cuestionario aporta una valoración de ocho dimensiones de la salud que son relevantes para la propia persona, como el bienestar emocional, la aceptación social, las relaciones en la familia, la participación, el entorno escolar, la autonomía, el dolor y el acceso a los servicios. Este artículo informa cómo se compone cada dimensión y los resultados de las pruebas de fiabilidad y validez de las escalas y de las puntuaciones que se producen a partir de su utilización.

## Introducción

La parálisis cerebral (PC) es un grupo de trastornos permanentes del desarrollo del movimiento y la postura ^1^ que causan limitación de la actividad y conllevan diversas alteraciones ^2^ . El impacto de la PC en la vida de las personas y su condición de irreversibilidad exige un abordaje acorde a las necesidades y el contexto de cada persona ^3^ , considerando diversas dimensiones de la salud y centrando la atención en la persona y su familia ^4^ .

La Clasificación Internacional del Funcionamiento, de la Discapacidad y la Salud (CIF) recomienda enfoques multidimensionales en la evaluación de las personas y tener en cuenta también aspectos sociales y contextuales, incorporando una perspectiva de derecho ^5^ . En este sentido, las medidas de salud informadas por la propia persona incorporan la valoración subjetiva de la salud, del estado funcional y bienestar en indicadores de múltiples dimensiones de la salud ^6^ y son apropiadas para la valoración de personas con discapacidad. Son mediciones complementarias a las clínicas, pueden dar cuenta de etapas significativas del desarrollo, del grado de afectación de su autonomía, así como de los resultados de intervenciones terapéuticas y de rehabilitación ^7-8^ .

Uno de los pocos instrumentos de evaluación de la calidad de vida relacionada con la salud (CVRS) específicos para niños, niñas y adolescentes (NNA) con PC es el cuestionario *Cerebral Palsy Quality of Life* (CP-QOL), que produce indicadores sobre el bienestar social y la aceptación, la participación, los sentimientos sobre el funcionamiento, el bienestar emocional, la autoestima y el dolor ^9^ . El CP-QOL fue desarrollado en Australia partiendo de entrevistas con NNA con PC, madres y padres ^10-11^ . Los procesos realizados condujeron a cuatro versiones, dos para autoinforme de 9 a 12 años (CP-QOL-Child) y de 13 a 18 años (CP-QOL-Teen); y dos para sus cuidadores principales (CP-QOL-PCQ-Child, 4-12 años; y CP-QOL-PCQ-Teen, 13-18 años, donde PCQ indica Primary Caregiver Questionnaire). Posteriormente, se publicó una versión española con las versiones autoinformadas unificadas para NNA de 8 a 18 años ^12^ .

En Argentina, las versiones para cuidadores fueron recientemente adaptadas siguiendo el método de traducción y retrotraducción por lingüistas profesionales, con control de la equivalencia por expertos y pruebas de comprensión mediante entrevistas cognitivas ^13^ . El objetivo del presente estudio fue lograr la estructura definitiva y evaluar la fiabilidad y validez del CP-QOL-PCQ en población argentina.

## Métodos

Estudio de diseño transversal en la población de madres, padres o cuidadores principales de NNA con diagnóstico de PC, residentes en Argentina. El protocolo siguió las recomendaciones del grupo desarrollador del CP-QOL ^13^ y fue aprobado por el Comité Institucional de Ética en la Investigación en Salud del Hospital Nacional de Clínicas, Universidad Nacional de Córdoba (UNC), con nota del 12/12/2017.

Inicialmente se convocó a 11 instituciones públicas y privadas de la provincia de Córdoba, de las cuales 7 colaboraron, siendo educativas y/o terapéuticas (centros de rehabilitación, de educación especial, etc.). Tras una interrupción debida a las medidas de control de la pandemia por SARS-COVID-19, se implementó un cuestionario digital con una convocatoria abierta a través de redes sociales, sumando respuestas desde diferentes provincias de Argentina. Una respuesta correspondiente a un joven de 24 años no fue excluida considerando que los patrones de desarrollo biológico y transiciones de roles sociales podrían ser similares a la adolescencia ^14^ .

En la primera etapa de la encuesta se utilizaron las versiones del CP-QOL equivalentes a las originales, con 88 ítems para adolescentes y 66 para niños y niñas. En la segunda etapa, tras un análisis preliminar, se aplicó una versión única con 70 ítems. El CP-QOL-PCQ se responde en una escala Likert de 9 opciones (1: muy inconforme; 9: muy conforme). El puntaje por dimensión se obtiene como promedio de las respuestas a los ítems de cada dimensión, de manera que valores altos representan niveles altos de CVRS. Los valores perdidos se reemplazaron por el promedio muestral.

Se implementó también el cuestionario KIDSCREEN-27 ^15-16^ , un instrumento genérico de CVRS que recoge información referida a Bienestar físico, Bienestar psicológico, Relación con padres y familia, Relación con pares, Entorno escolar y un Índice general de CVRS.

Se registraron datos del NNA (edad, sexo, nivel de funcionalidad según el Gross Motor Function Classification System -GMFCS- y cobertura de salud) y de la persona que respondió (relación con el NNA) y de la madre del NNA (nivel de estudios, informado por quien respondió el cuestionario). El valor de GMFCS fue provisto por los profesionales de salud de los centros de rehabilitación, pero no se pudo obtener en los casos contactados a través de un centro escolar.

El análisis de datos siguió objetivos específicos: (a) describir la muestra usando medias (M) y desvíos estándar (DE) o proporciones (%); (b) informar la distribución estadística de cada ítem con valores mínimo, máximo, media, desviación estándar, asimetría y curtosis; (c) establecer la estructura y consistencia interna mediante una estrategia que tuvo como referencias teórico-empíricas los procesos de desarrollo de las versiones originales ^11 13^ y de la versión española ^12^ , controlando correlaciones inter-ítems (>0.30), alfa de Cronbach (>0.70), y la distribución de cada dimensión resultante, incluyendo el efecto suelo y techo (aceptables hasta un 15% de casos en los valores extremos ^17^ ); (d) corroborar la convergencia de las escalas del CP-QOL-PCQ con dimensiones similares del KIDSCREEN, estableciendo hipótesis a priori y calculando correlaciones que se esperaban al menos moderadas (r>0.30); y (e) contrastar las dimensiones evaluadas por grupos hipotéticamente diferentes como evidencia de validez basada en grupos conocidos, para lo cual se compararon las puntuaciones medias de las escalas del CP-QOL-PCQ según edad (niños y niñas versus adolescentes) y grado de afectación motora de acuerdo con el GMFCS (leve/moderada versus grave). En este último paso, se realizó la prueba t (p< 0,05) y la diferencia de medias estandarizadas (d de Cohen), estableciendo que las diferencias mayores a 0,2 dan cuenta de diferencias significativas ^18 8^ . Los análisis estadísticos se realizaron con el programa SPSS 24.

## Resultados

Se consiguió información de 100 NNA de 3 a 24 años (M=13,3; DE=4,9), de los que el 53,5% fueron varones, el 37,6% tenía afectaciones motoras graves (niveles 4 y 5 en GMFCS), el 31,7% tenía cobertura sanitaria pública solamente y el 87,1% residía en la Provincia de Córdoba). La edad media de quienes respondieron fue 42,7 años (DE=9,5), la mayoría fueron madres (83,1%) y el 44% tenía secundario incompleto o menor nivel de escolaridad (Tabla 1).

**Tabla N° 1:** Características sociodemográficas y clínicas de la muestra

	f	%
Edad del/la NNA		
Niño/a (4 a 12 años)	45	44,6
Adolescente (13 a 18 años)	42	41,6
Joven (19 a 24 años)	13	13,8
Sexo		
Varón	54	53,5
Mujer	46	45,5
No Responde	1	1,0
GMFCS		
Nivel 1	8	7,9
Nivel 2	12	11,9
Nivel 3	11	10,9
Nivel 4	20	19,8
Nivel 5	18	17,8
Valores perdidos (*)	31	31,7
Cobertura de salud declarada		
Privada	66	65,3
Solo pública	32	31,7
Valores perdidos	2	3,0
Provincia de residencia		
Córdoba	87	87,0
Otras Provincias	13	13,0
Relación de quien responde con el/la NNA		
Madre	84	83,1
Padre	9	8,9
Otro familiar	7	7,0
Nivel máximo de estudios de la madre		
Baja (secundario incompleto)	44	44,0
Media (terciario o universitario incompleto)	29	29,0
Alta (terciario o universitario completo)	27	27,0

(*) En los casos contactados en centros escolares las madres, padres y otros cuidadores no conocían este dato.

La distribución estadística de cada ítem reveló, en general, suficiente variación con valores en todo el rango de puntuación posible (1-9), medidas de tendencia central y dispersión globales en torno al centro del rango (M = 5,98, DE = 1,89) y una aceptable distribución evaluada por la asimetría (<3,0) y curtosis (<7,0) de los ítems (datos disponibles; no incluidos en tablas). Como resultado del análisis de ítems y de estructura y consistencia interna de las escalas, se optó por la conformación de ocho escalas que lograban resultados psicométricos satisfactorios (tabla 2), cuyo contenido se presenta en el Cuadro 1. Las distribuciones de las puntuaciones de las escalas logran valores aceptables de normalidad, correlaciones inter-ítems satisfactorias (<0.30) y buena consistencia interna (>0.70). Solo la dimensión Relaciones en la familia mostró efecto techo (38%).

**Cuadro N° 1:** Síntesis del contenido por dimensiones de la CVRS del CP QOL PCQ

Modalidad de la pregunta: El CP QOL PCQ pregunta a la persona que responde “cómo piensa que el chico o chica se siente con respecto a…”
Dimensión y aspectos relevados por sus ítems Bienestar emocional: Sentimientos sobre él/ella mismo/a, sus motivaciones, sus oportunidades en la vida, su capacidad para hacer las cosas que quiere hacer y su aspecto físico.
Bienestar y aceptación social: Sentimientos acerca de cómo se lleva y cómo es aceptado por la gente en general, por adultos y por otros chicos fuera de la escuela o colegio. Cómo se siente al juntarse con amigos, sobre la forma en que intenta probar cosas nuevas y sobre la manera en que se comunica con la gente.
Relaciones en la familia: Sentimientos respecto al apoyo que tiene de su familia, la aceptación por su familia y a salir de viaje con la familia.
Participación: Sentimientos con respecto a su capacidad para participar en actividades recreativas y de tiempo libre, en actividades deportivas, en eventos sociales fuera de la escuela o colegio y en su comunidad.
Entorno escolar: Sentimientos acerca de cómo se lleva con otros chicos en la escuela o colegio y con sus maestros, profesores y/o asistentes, cómo es aceptado/a por otros estudiantes y por el personal. Si se siente tratado/a de la misma manera que los demás y sobre su capacidad para participar en la escuela o colegio.
Autonomía: Sentimientos sobre su capacidad para moverse en su barrio, para ir de un lugar a otro, para vestirse solo/a, para beber sin ayuda, para ir al baño sin ayuda. Acerca de hacer cosas solo/a, sin compañía y sin depender de otros, así como su forma de usar sus piernas, sus brazos y sus manos.
Dolor: Salud general, sensación acerca de cómo duerme, cuánto dolor siente, qué nivel de incomodidad siente. Sentimientos acerca de la forma en que los dolores le afectan en su vida, le impide ser él/ella mismo/a o no le permite pasarlo bien todos los días.
Acceso a servicios: Esta dimensión se indaga cómo se siente la persona que responde respecto del acceso del chico/a al tratamiento, a atención médica o quirúrgica especializada, a su capacidad para recibir asesoramiento de un pediatra, al acceso a ayuda adicional de aprendizaje dentro del colegio y al acceso a terapias como fisioterapia, fonoaudiología o terapia ocupacional.

**Tabla N° 2:** Estructura interna y consistencia interna del CP QOL-PCQ

Dimensión	N de ítems	M (DE)	g1	g2	λM	α	Efecto suelo	Efecto techo
Bienestar emocional	5	6,47 (1,44)	-0,43	-0,2	0,49	0,83	0 %	3 %
Bienestar y aceptación social	12	6,70 (1,17)	-0,47	0,67	0,44	0,89	0 %	1 %
Relaciones en la familia	3	8,11 (1,09)	-1,56	2,27	0,54	0,78	0 %	38 %
Participación	4	6,74 (1,49)	-0,08	-0,96	0,54	0,82	0 %	11 %
Entorno escolar	7	7,47 (1,08)	-0,54	-0,28	0,61	0,91	0 %	10 %
Autonomía	10	5,47 (1,49)	-0,23	-0,22	0,37	0,85	0 %	0 %
Dolor	8	6,89 (1,89)	-0,77	-0,4	0,65	0,94	0 %	6 %
Acceso a servicios	5	7,25 (1,24)	-1,02	1,96	0,52	0,84	0 %	8 %

Nota: λM: correlación inter-ítems promedio; α: coeficiente alfa de Cronbach; g1: Asimetría; g2: Curtosis.

En cuanto a la validez convergente, las correlaciones entre las dimensiones del CP-QOL-PCQ y del KIDSCREEN que miden conceptos similares resultaron moderadas y altas, como Bienestar emocional y Bienestar psicológico, Relaciones en la familia y vida familiar, y Entorno escolar de ambos cuestionarios. Además, todas las dimensiones del CP-QOL-PCQ se correlacionaron adecuadamente con el índice global de CVRS del KIDSCREEN (Tabla 3).

**Tabla N° 3:** Correlaciones entre CP QOL-PCQ y KIDSCREEN-27

Dimensiones de la CVRS	KIDSCREEN					
CP-QOL	Bienestar físico	Bienestar psicológico	Vida familiar	Amigos	Entorno escolar	Índice de CVRS
Bienestar emocional	0,46**	0,46**	0,32**	0,26*	0,49**	0,53**
Bienestar y aceptación social	0,28*	0,34**	0,40**	0,36**	0,52**	0,51**
Relaciones en la familia	0,16	0,43**	0,49**	0,41**	0,47**	0,44**
Participación	0,41**	0,43**	0,40**	0,35**	0,52**	0,61**
Entorno escolar	0,24*	0,31**	0,23*	0,13	0,60**	0,38**
Autonomía	0,35**	0,26**	0,26*	0,18	0,38**	0,26*
Dolor	0,21*	0,25*	0,18	0,03	0,26*	0,28*
Acceso a servicios	0,18	0,30**	0,21	0,15	0,37**	0,26*

Nota. * p < .05, ** p < .01.

Las pruebas de hipótesis de validez basada en grupos conocidos fueron corroboradas en la mayoría de las dimensiones (d>0,20) al observar, en general, que el grupo de niños y niñas tuvo puntuaciones promedio más altos que el grupo de adolescentes (Tabla 4) y que quienes tenían una afectación grave de la funcionalidad puntuaron más bajo que quienes tenían una afectación leve o moderada (Tabla 5).

**Tabla N°4:** Diferencias de medias del CP QOL-PCQ según edad.

Edad	4 a 12 años	13 a 24 años		
	M (DE)	M	T (p)	D
Bienestar emocional	6,43 (1,24)	6,49 (1,59)	-0,20 (0,83)	0,04
Bienestar y aceptación social	6,73 (1,05)	6,69 (1,27)	0,17 (0,86)	0,03
Relaciones en la familia	8,40 (0,71)	7,88 (1,28)	2,38 (0,01)	0,27
Participación	6,52 (1,37)	6,91 (1,52)	-1,28 (0,20)	0,26
Entorno escolar	7,53 (1,03)	7,38 (1,21)	0,47 (0,63)	0,09
Autonomía	5,71 (1,34)	5,27 (1,59)	1,47 (0,14)	0,29
Dolor	6,67 (1,84)	7,06 (1,93)	-1,02 (0,30)	0,2
Bienestar emocional	7,47 (1,07)	7,07 (1,34)	1,63 (0,10)	0,32

Nota: M: media; DE: desviación estándar; T= prueba t; p= nivel de significación; D= tamaño del efecto (d de Cohen), donde 0,2-0,5 son diferencias pequeñas, 0,51-0,8 son diferencias moderadas y >0,8 son diferencias grandes.

**Tabla N° 5:** Diferencias de medias del CP QOL-PCQ según nivel de funcionalidad

Funcionalidad	Leve-Moderado	Grave		
	M (DE)	M	T (p)	D
Bienestar emocional	6,34 (1,22)	6,03 (1,59)	0,92 (0,36)	0,22
Bienestar y aceptación social	6,54 (0,94)	6,46 (1,28)	0,25 (0,79)	0,07
Relaciones en la familia	7,90 (1,00)	7,94 (1,35)	-0,13 (0,89)	0,02
Participación	6,82 (1,33)	6,29 (1,55)	1,48 (0,14)	0,36
Entorno escolar	7,12 (0,91)	7,36 (1,24)	-0,91 (0,36)	0,22
Autonomía	5,61 (1,46)	5,01 (1,63)	1,55 (0,12)	0,38
Dolor	7,47 (1,42)	6,39 (2,27)	-2,25 (0,03)	0,57
Bienestar emocional	6,99 (1,05)	7,27 (1,48)	-0,89 (0,37)	0,21

Nota: M: media; DE: desviación estándar; T= prueba t; p= nivel de significación; D= tamaño del efecto (d de Cohen), donde 0,2-0,5 son diferencias pequeñas, 0,51-0,8 son diferencias moderadas y >0,8 son diferencias grandes.

## Discusión

Este trabajo presenta la versión argentina del CP-QOL-PCQ para medir CVRS en población infantil y adolescente con PC, con propiedades psicométricas satisfactorias en siete escalas o dimensiones de la CVRS y una de valoración del acceso a servicios sanitarios según la perspectiva del cuidador principal.

Esta versión tiene algunas diferencias con las versiones previas, de Australia y España. En el proceso de adaptación se descartaron ítems que no lograron equivalencia en el contexto argentino y otros que revelaron problemas de comprensión en pruebas cognitivas en población diana ^13^ . Durante los análisis estadísticos se excluyeron ítems con alto porcentaje de valores perdidos, deficiente distribución de respuestas o que no correlacionaran adecuadamente a una dimensión en función del concepto al que se esperaba que aportaran. Las versiones australianas para informadores indirectos cuentan con 65 (niños/as) y 88 ítems (adolescentes), además de diferencias en su estructura multidimesional, como resultado de procesos realizados por separado. Por su parte, la versión española resultó en un único cuestionario de 53 ítems ^12^ , lo que presenta la ventaja de poder conformar grupos de mayor tamaño en y comparar casos en un rango de edades mayor con los mismos indicadores o dimensiones. La versión argentina se compone de 54 ítems y guarda mayor equivalencia con la versión española, excepto en que la versión argentina permite computar las dimensiones de Bienestar emocional, Aceptación social y Participación, que en la versión española conforman una única dimensión. Esta decisión se basó en el reconocimiento de conceptos específicos, que evalúan aspectos de interés diferente en el impacto que la PC tiene en la calidad de vida de NNA, así como en los buenos resultados estadísticos de las tres escalas. Otra diferencia con las versiones australianas y española es que descartamos la dimensión complementaria de salud de familiares, porque no alcanzó niveles aceptables de correlación entre ítems como para computar una escala y no representa una dimensión teórica de la CVRS de NNA.

Los resultados de consistencia interna de las escalas fueron muy buenos, algo superiores a los las escalas de las versiones originales australianas. Una limitación métrica a tener en cuenta es la acumulación de respuestas en los valores más altos en la escala de Relaciones en la familia (efecto techo), que puede limitar la discriminación de este indicador en futuras aplicaciones. Quizás esto se debe a que es un familiar quien responde el cuestionario, lo cual podría superarse con versiones de autorreporte.

Como pruebas de validez convergente, los dominios de CP-QOL-PCQ se correlacionaron adecuadamente con los dominios de KIDSCREEN-27 con conceptos similares, coincidiendo con los estudios precedentes ^11-12^ . Otra evidencia de validez se obtuvo al corroborar que las escalas del CP-QOL-PCQ puntúan más bajo ante situaciones que se sabe previamente que deterioran la CVRS, como la transición de la niñez a la adolescencia ^19-20^ o el agravamiento de las complicaciones funcionales propias de la PC ^21^ .

Las recomendaciones internacionales sobre instrumentos para medir salud o resultados percibidos por pacientes, dentro de los cuales la CVRS es uno de los principales, señalan una serie de criterios sobre los que se debe disponer de información y resultados satisfactorios ^22-23^ . Este trabajo ofrece información sobre el modelo conceptual que mide este cuestionario, así como resultados aceptables de fiabilidad, validez y carga de administración. El proceso de adecuación cultural y lingüística fue presentado previamente ^13^ y es una de las fortalezas de este cuestionario al ofrecer una alta equivalencia semántica, adecuación cultural a nuestro contexto y probada aceptabilidad lingüística a la población local, como se recomienda para este tipo de medidas ^24^ . En este trabajo implementamos diversas modalidades de administración, iniciando con entrevistas, y posteriormente autoadministración en papel y en plataforma digital, con un tiempo de aplicación que ronda los 25 minutos, lo que significa una carga de administración aceptable.

Una de las limitaciones del estudio fue el tamaño de la muestra debido a dificultades para contactar potenciales participantes y un 50% de respuesta lograda, problemas atribuibles a la fragmentación de nuestro sistema sanitario, a las dificultades que afrontan las familias frente al cuidado de una persona con discapacidad y a la coyuntura epidemiológica que impuso la pandemia de COVID-19. Por ello también fue inviable implementar una estrategia longitudinal para evaluar la confiabilidad test-retest y la sensibilidad a los cambios. Futuros estudios podrían abordar esa evaluación longitudinal de mediano y largo plazo para ofrecer garantías para la valoración del impacto de abordajes terapéuticos a lo largo del tiempo, independientemente de la edad cronológica.

Un desafío en esta línea de investigación es lograr una versión del CP-QOL para autorreporte de los propios NNA con PC, que permita conocer sus experiencias directamente y, si acaso, analizar el grado concordancia o diferencias con la mirada de sus cuidadores ^25^ . En la práctica clínica o en el ámbito escolar, este instrumento puede informar el impacto de la PC y de múltiples determinantes de la salud en la población que padece este trastorno, siendo un indicador útil para la valoración de programas terapéuticos de abordaje integral y multidimensional de la salud.

## Conclusión

Este trabajo presenta la versión del CP-QOL-PCQ para ser aplicado en NNA con PC en Argentina, con un modelo conceptual similar a las versiones de referencia, conformado por 54 ítems que ofrecen información multidimensional en 8 escalas, de las cuales se proporciona evidencias de fiabilidad y validez en los niveles esperados para un instrumento de medición de la CVRS.
